
JOURNAL INFORMATION
==============================
NLM Title Abbreviation: ScientificWorldJournal
Journal ID: ScientificWorldJournal
NLM Journal ID: 101131163
Journal ID: TSWJ

The Scientific World Journal

ISSN: 2356-6140
EISSN: 1537-744X
Publisher: Wiley

ARTICLE INFORMATION
==============================
PMCID: PMC6084552
PMID: 30147474
DOI: 10.1100/tsw.2001.159
Article ID: 487262
Article version: 1
Subjects: Short Report

Activation of the JNK/SAPK and P38 Mitogen-Activated Protein Kinase Signaling Pathways Sensitize Tumor Cells to Cisplatin-Induced Apoptosis

Mansouri Abdel
Ridgway Lon D.
Zhang Qingxiu
Claret Francois X. *
The University of Texas-M.D. Anderson Cancer Center, Dept. of Molecular Therapeutics, 1515 Holcombe Blvd., Box 317, Houston, Texas 77030, USA
*Francois X. Claret: fxclaret@mdanderson.org
Publication date: 2001
Electronic publication date: 2001 Dec 12
Volume: 1
First page: 35
Last page: 35
Copyright: Copyright © 2001 Abdel Mansouri et al.
Copyright year: 2001
License: This is an open access article distributed under the Creative Commons Attribution License, which permits unrestricted use, distribution, and reproduction in any medium, provided the original work is properly cited.
License URL: https://creativecommons.org/licenses/by/3.0/

==============================
Miami *Nature Biotechnology* Short Reports
*TheScientificWorld* (2001) 1(S3), 35SR
ISSN 1532-2246; DOI 10.1100/tsw.2001.159

### ACTIVATION OF THE JNK/SAPK AND P38 MITOGEN-ACTIVATED PROTEIN KINASE SIGNALING PATHWAYS SENSITIZE TUMOR CELLS TO CISPLATIN-INDUCED APOPTOSIS

Abdel Mansouri, Lon D. Ridgway, Qingxiu Zhang, and Francois X. Claret*

The University of Texas-M.D. Anderson Cancer Center, Dept. of Molecular Therapeutics, 1515 Holcombe Blvd., Box 317, Houston, Texas 77030, US
* fxclaret@mdanderson.org

**INTRODUCTION.** Chemoresistance is a major impediment to the successful treatment of cancer. However, the mechanisms of chemoresistance are poorly understood. Cisplatin (CDDP) treatment activates multiple signal transduction pathways that can lead to diverse cellular responses, including cell cycle arrest (G2/M), DNA repair, or apoptosis. Differences in early signal transduction activity in response to chemotherapy could provide insight regarding the ultimate outcome of treatment. In this study, we attempted to elucidate the mechanisms of resistance to cisplatin in a cisplatin-sensitive human ovarian cancer cell line and a cisplatin-resistant clone (2008 and 2008.C13 respectively).

**METHOD.** We analyzed the relative activation of 3 major MAPK signaling pathways: p38, JNK/SAPK, and ERK1/2. We also analyzed the differential expression of pro-apoptotic and anti-apoptotic-related genes in response to cisplatin treatment in our isogenic cell line model.

**RESULTS.** There was a prolonged and persistence activation (up to 12 h) of the p38 and JNK/SAPK pathways in response to cisplatin in the sensitive cells (2008). In contrast, the cisplatin-resistant clone (2008.C13) displayed a shorter duration of activation (1-3 h) of the p38 and JNK/SAPK pathways. The ERK pathway was activated to a similar extent at the early time points in both cell lines. Cisplatin-induced apoptosis was associated with cytochrome c release and subsequent caspase-3 activation and PARP cleavages. We found that inhibition of the JNK/SAPK and p38 pathways by small drug inhibitors mediate a resistant phenotype to cisplatin and increase survival. Moreover, 3T3 cells with disrupted *c-jun* (3T3 cJun$^{-/-}$) were more resistant to cisplatin cell death than the parental 3T3 cells. Our results suggest that in cisplatin-treated cells, induction of apoptosis can take place as a consequence of JNK activation. This indicate that a critical difference in sensitivity *versus* drug-resistance is associated with in duration of the activation of the p38 and JNK/SAPK signal transduction pathways and their downstream target genes (e.g. AP-1 target genes), in response to genotoxic agents (1-4).

Our results are consistent with the hypothesis that prolonged activation of the JNK/SAPK and p38 pathways in response to cisplatin treatment of ovarian cancer cells leads to cell death (1,5). Early signal transduction events probably indicate and/or dictate whether the cell will undergo cell division or apoptosis. Cisplatin resistance may be related to attenuation of certain MAPK signals. Taken together, our findings suggest that duration of JNK activation and p38 play an active role in mediating cisplatin-induced apoptosis in ovarian carcinoma cells and functions upstream of caspase activation to initiate the apoptotic signal.

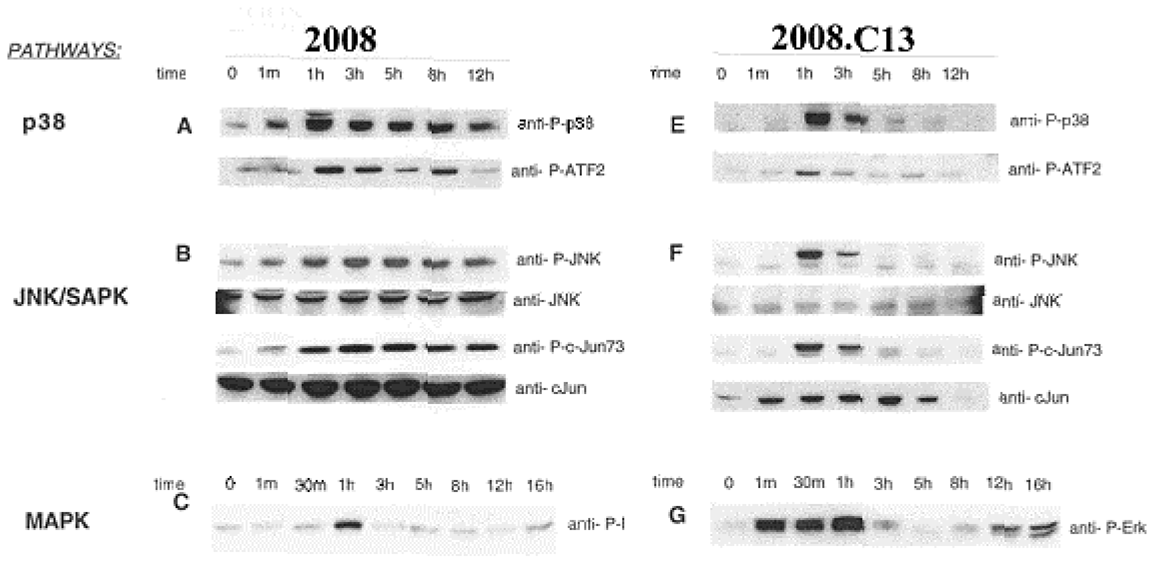

**Fig. 1.** MAPK Signal Transduction Pathways Activation in Response to Cisplatin Treatment in Ovarian Cancer Cell Lines 2008 and 2008.C13.

### REFERENCES.

1. Potapova, O., Haghighi, A., Bost, F., Liu, C., Birrer, M.J., Gjerset, R., and Mercola, D. (1997) J. Biol. Chem. 272, 14041-14044
2. Le-Niculescu, H., Bonfoco, E., Kasuya, Y., Claret, F.X., Green, D.R., and Karin, M. (1999) Mol. Cell. Biol. 19, 751-763
3. Kolbus, A., Herr, I., Schreiber, M., Debatin, K.M., Wagner, E.F., and Angel, P. (2000) Mol. Cell. Biol. 20, 575-582
4. Eichhorst, S.T., Muller, M., Li-Weber, M., Schulze-Bergkamen, H., Angel, P., and Krammer, P.H. (2000) Mol. Cell. Biol. 20, 7826-7837
5. Sanchez-Perez, I., Murgia, J.R., and Perona, R. (1998) Oncogene 16, 533-546
